# Radiographic predictors for MRONJ in oncologic patients undergoing tooth extraction

**DOI:** 10.1038/s41598-022-15254-y

**Published:** 2022-07-04

**Authors:** Catalina Moreno-Rabié, Laurence Lapauw, Hugo Gaêta-Araujo, André Ferreira-Leite, Wim Coucke, Tim van den Wyngaert, Reinhilde Jacobs

**Affiliations:** 1 OMFS-IMPATH Research Group, Department of Imaging and Pathology, Faculty of Medicine, University of Leuven, Leuven, Belgium; 2 Department of Oral and Maxillofacial Surgery, University Hospitals Leuven, Leuven, Belgium; 3 Oral Radiology Area, School of Dentistry, Federal University of Alfenas, Alfenas, Brazil; 4 Department of Dentistry, Faculty of Health Sciences, University of Brasília, Brasilia, Brazil; 5 Certified Freelance Statistician, Heverlee, Belgium; 6 Department of Nuclear Medicine, Antwerp University Hospital, Edegem, Belgium; 7 Faculty of Medicine and Health Sciences, University of Antwerp, Antwerp, Belgium; 8 Department of Dental Medicine, Karolinska Institutet, Stockholm, Sweden

**Keywords:** Oral diseases, Risk factors, Oral manifestations

## Abstract

Tooth extraction is a risk factor for the development of osteonecrosis of the jaw following treatment with antiresorptive drugs (ARDs), but not all extraction sites develop this pathology. Therefore, we aimed to identify local radiographic predictors of Medication-Related Osteonecrosis of the Jaw (MRONJ) in panoramic images of oncologic patients undergoing tooth extraction. Based on a retrospective longitudinal cohort study design, patients were included if undergoing one or more tooth extraction, with at least one administration of ARDs, and presence of pre- and post-operative panoramic radiographs. After data collection, blinded and independent observations were performed. Eleven distinct imaging-related parameters were assessed preoperatively and five postoperatively, at each extraction site. A case–control and subgroup analysis assessing MRONJ development was performed. Significance level is set to 0.05 (5%). A total of 77 oncologic patients were selected, undergoing 218 tooth extractions, from which 63 teeth (29%) in 39 patients (51%) developed MRONJ. Results showed that patients developed significantly more MRONJ with longer ARD treatment (*p* = 0.057), teeth with absent and incomplete endodontic fillings with caries, widened periodontal ligament space and/or periapical lesions (*p* = 0.005), and sclerotic and heterogenous bone patterns (*p* = 0.005). In conclusion, tooth extraction sites presenting with infections and bone sclerosis are at higher risk to develop MRONJ.

## Introduction

Antiresorptive drugs (ARDs) are widely used as treatment of patients with osteoporosis and cancer, among other diseases^1^. Particularly in an oncologic setting, these drugs are used to effectively prevent skeletal morbidity in patients with metastatic bone disease or multiple myeloma^2^, which may involve pain, nerve compression, and pathologic fractures^3^. Despite the benefits of ARDs, a common adverse effect is Medication-Related Osteonecrosis of the Jaws (MRONJ)^1,4^.

MRONJ corresponds to exposed bone or bone that can be probed through an intraoral or extraoral fistula(e) in the maxillofacial region that has persisted for more than eight weeks, in patients treated with antiresorptive drugs and who have not received radiation therapy to the head and neck region nor have obvious metastatic disease in the jaws^5^. Besides the stages involving bone exposure, the American Association of Maxillofacial Surgeons (AAOMS) proposed two additional groups, namely "at risk" and stage 0. Whereas both refer to the absence of bone exposure, the first includes asymptomatic patients receiving ARD and the second one involves patients presenting with non-specific symptoms or clinical and radiographic findings^6^.

Within the systemic risk factors associated to MRONJ development are the type and dose of these medications, but most importantly their cumulative dosage, in particular the higher doses administered for longer periods^7,8^. Some examples of bisphosphonates are zoledronic acid, alendronate, ibandronate, pamidronate, and risedronate. Other implicated drugs include monoclonal antibodies such as denosumab^9^, which, like bisphosphonates, alter the bone resorption-apposition balance to prevent skeletal-related events.

Tooth extractions are often reported as an important triggering factor for this pathology. Though, experts suggest that underlying infections at the extraction site, such as periodontitis or periapical lesions, could play an even greater role in the onset of MRONJ^7,10^, especially when analyzing patients who had multiple tooth extractions and in whom only some of these sites developed osteonecrosis^11^, thus having the same systemic condition, but different local factors. Perhaps these sites could be masking an unexposed form of the pathology that would only be revealed at the time of extraction^7^, presenting in their radiographic appearance, sclerosis, thickening of the lamina dura, and persistence of the extraction socket^12^. In these cases, a radiographic evaluation is of great value to identify hidden lesions or abnormal bone patterns and recognize the high-risk sites for MRONJ.

Bearing the previous evidence in mind, the main objective of this study was to identify local radiographic characteristics in panoramic images that act as a risk factor for development of MRONJ in oncologic patients currently taking or with a history of antiresorptive drugs undergoing tooth extractions.

## Material and methods

### Study design and settings

Prior to the start of this study, ethical approval was granted by the ethical committee of UZ/KU Leuven (S63934) and waived the need for informed consent. This study corresponds to a retrospective longitudinal cohort study, and to report the present information, the STROBE guidelines were followed^13^. In addition, ethical standards from the Institutional Review Board and the Helsinki Declaration were obeyed.

### Participant selection

A retrospective search was carried out in the database of University Hospitals Leuven, where medical files of patients in treatment with ARD, who visited the Oral and Maxillofacial Surgery department between January 1st, 2010, and December 30th, 2019, were assessed. The inclusion criteria included, (1) patients in the category “at risk” according to the AAOMS^6^, (2) at least one administration of ARD in oncologic doses, (3) had undergone one or more tooth extractions, (4) had a pre- and post-operative panoramic image, and (5) documented follow-up until mucosal healing within eight weeks to refute or exposed bone for at least eight weeks to confirm the clinical development of MRONJ. Exclusion criteria were, (1) history of radiation in the head and the neck region, (2) prior MRONJ diagnosis, (3) insufficient image quality (i.e., artefacts) and/or objects in the extraction site (i.e. implants), (4) pre-operative radiographs acquired earlier than one year prior to tooth extraction, and (5) concomitant maxillofacial pathologies.

After including the study group, a control group of patients who were age-, gender-, tooth-, and sextant of extraction matched was selected from the same database. Inclusion criteria for the control group were, no intake of ARD and having panoramic radiographs from before and after a tooth extraction. Further exclusion criteria were as mentioned for the study patients.

### Data selection

Along with the panoramic images, clinical information was collected from the patient’s files. The following variables were included: age, gender, tobacco and alcohol use, oncologic diagnosis, previous chemotherapy and/or radiotherapy to other body regions than to the head and neck, antiresorptive drug, dosage, treatment duration, corticosteroids intake, date of the tooth extraction(s), number, site and extracted tooth, date of pre- and post-operative panoramic radiograph, development of MRONJ, date of diagnosis, staging according to the AAOMS^6^, site of development, presence of drug holiday at least 60 days before the extraction, use of leukocyte- and platelet-rich fibrin (L-PRF), prophylactic antibiotics, and antiseptic mouthwash.

### Radiographic assessment

Panoramic radiographs were acquired using VistaPano S or S Ceph (Dürr Dental SE, Bietigheim-Bissingen, Germany) at 73 kVp, 12 mA, and an exposure time of 7 s. Images of eligible study and control participants were anonymized, exported in DICOM format, and assigned an arbitrary participant number. All images were then transformed to TIFF files, and observations were done using Image J program version 1.53j (Wayne Rasband, https://imagej.nih.gov/ij/). Blinded and independent observations were performed by two dentomaxillofacial radiologists and a general dentist. Prior to the radiographic assessment, a calibration session was held to assess 20 panoramic images from ten before and after tooth extraction cases external to this study, to achieve baseline consensus in the diagnosis. The observations took place in a room with dim light, using a high-resolution display (MD Barco MDRC-2221; Barco, Kortrijk, Belgium) at approximately 60 cm. After evaluation, the result of the observation was calculated using the mode. For example, if two observers judged a parameter as present and one as absent, then it was considered as present. If the mode could not be applied because three different interpretations were assigned, the case was discussed individually until agreement was reached. One month after the first assessment, a second reading session was carried out with 10% of the sample to evaluate the intraobserver agreement.

The following parameters were assessed in the panoramic radiographs before the extraction at a tooth level, based on the description of Gaêta-Araujo et al.^11^:

1. Horizontal bone loss: absent/initial bone loss, if bone resorption was up to 1/3 cervical of the root, or moderate/severe bone loss, if bone resorption was more than 1/3 cervical of the root.
2. Angular bone loss: absent or present.
3. Furcation involvement: absent or present.
4. Periodontal ligament space: normal or widened.
5. Lamina dura: normal or thickened.
6. Root remnant: absent or present.
7. Periapical lesion: absent or present.
8. Endodontic treatment: absent, adequate endodontic filling in length or width, or inadequate endodontic filling (over and underfilling).
9. Prosthodontics: absence or presence of crowns, bridges, and/or fillings.
10. Caries: absent, dentin caries, or caries in contact with or overlapping the pulp cavity.
11. Bone pattern surrounding the tooth: normal, sclerotic (increased radio-opacity), radiolucent, or heterogeneous (mixed radiolucent and radiopaque).

Furthermore, parameters appraised in the extraction site on the respective post-operative panoramic image included:

1. Bone pattern: as described before.
2. Alveolar socket: not visible or visible.
3. Lamina dura: not visible or visible.
4. Sequestrum formation: absent or present.
5. Crater-like defect: with reference to an accentuated resorption in the form of a prominent concavity, absent or present.

### Statistical analysis

The statistical analysis was done using RStudio Software version 4.0.4 (RStudio, Boston, MA US). A *p*-value ≤ 0.05 was considered statistically significant. Fleiss’ Kappa test was used to calculate interobserver agreement and Cohen’s Kappa test for intraobserver agreement. Agreement was considered fair when the test result was > 0.21–0.40, moderate when > 0.41–0.60, substantial when > 0.61–0.80, and almost perfect when > 0.81–0.99^14^.

#### Univariate analysis

The significance of differences in characteristics between control and study patients was tested before the start of the analysis for gender, age, and extracted tooth, using Chi-Square and Wilcoxon rank-sum test.

Observations and clinical data documented for each extracted tooth were tested for independency using the Chi-square/Fisher’s exact test and the Wilcoxon rank-sum test for ordinal variables. Comparisons were made between the control and antiresorptive-treated group. In addition, the latter was divided into sites that developed MRONJ (MRONJ+) after tooth extraction and sites that did not (MRONJ−). The null hypothesis was that the assessed parameters are independent in the control and study group, and in the MRONJ+ and MRONJ− extraction sites. Lastly, the McNemar-Bowker test was used to compare pre- and post-operative appearance of bone pattern.

Considering that some patients had multiple tooth extractions, with some sites MRONJ+ and other sites MRONJ−, further analysis was carried out using a generalized linear mixed model, which tested the independency of the assessed variables while respecting the grouped character of the data.

#### Multivariate analysis of risk factors

A stepwise model selection was performed, through a generalized linear model for binary data using the logit link and with patient as random factor, to identify the combination of variables that had the best relationship with MRONJ development among the study group. The assessed variables were horizontal bone loss, angular bone defect, furcation involvement, periodontal ligament, lamina dura, root remnant, periapical lesion, endodontic treatment, presence of composite or crown, presence of caries, pre-operative bone pattern, type of extracted tooth, sextant, duration of ARD treatment, and presence of drug holiday. For those variables that were part of the selected model, a pairwise comparison between the group's variables was performed and corrected for simultaneous hypothesis testing according to Tukey.

## Results

In this ten-year observational study, 1468 patients visited the Oral and Maxillofacial Surgery department and were currently or in the past treated with ARDs. From these patients, 927 had either prior diagnosis of MRONJ or only one panoramic image available, 219 patients did not have tooth extraction, 130 received ARD treatment for other reasons than oncology, 89 did not have either a pre- or post-operative panoramic image, 22 had images with bad quality, and 4 had panoramic images older than one year before their tooth extraction.

From the total, 77 patients, who underwent 218 tooth extractions complied with the inclusion criteria, and 88 patients with 238 tooth extractions were selected as controls. The study and control group showed no significant differences regarding age (W = 3392, *p* = 0.992), gender (X^2^ = 0.0185, *p* = 0.892), tooth (W = 26,859, *p* = 0.514), sextant (W = 25,718, *p* = 0.872), and number of extractions (W = 3293, *p* = 0.739). Demographic data can be found in Table 1, at a patient level, and in Table 2, at a tooth level.

**Table 1:** Descriptive data from study group and control patients.

Characteristics	Oncologic group				Control
Number of patients, n	77				88
Development of osteonecrosis, n	MRONJ+	%	MRONJ−	%	NA
	39		38		
Age a tooth extraction (mean ± SD)	68.4 ± 11.3		67.2 ± 10.9		67.9 ± 11.2
**Age (years)**					
30–45	1	50%	1	50%	3
46–60	11	52%	10	48%	22
61–75	12	40%	18	60%	43
76–92	15	63%	9	38%	20
**Sex, n**					
Female	19	45%	23	55%	50
Male	20	57%	15	43%	38
**Underlying disease, n**					
Breast cancer	17	49%	18	51%	NA
Prostate cancer	9	60%	6	40%	NA
Multiple myeloma	6	33%	12	67%	NA
Lung cancer	3	60%	2	40%	NA
Gastrointestinal cancer	1	100%	0	0%	NA
Renal cancer	3	100%	0	0%	NA
**Chemo- and radiotherapy, n**					
None	4	50%	4	50%	NA
Chemotherapy	6	67%	3	33%	NA
Radiotherapy	7	58%	5	42%	NA
Both	22	46%	26	54%	NA
**Antiresorptive drug, n**					
Bisphosphonate	12	41%	17	59%	NA
Denosumab	19	51%	18	49%	NA
Both	8	73%	3	27%	NA
**Number of ARDs, n**					
1	31	48%	33	52%	NA
2	7	58%	5	42%	NA
3	1	100%	0	0%	NA
**Time on ARDs (months), n (%)**					
≤ 12	12	41%	17	59%	NA
> 12– ≤ 24	10	40%	15	60%	NA
> 24– ≤ 36	11	79%	3	21%	NA
> 36– ≤ 48	1	33%	2	67%	NA
> 48– ≤ 60	4	80%	1	20%	NA
> 60– ≤ 120	1	100%	0	0%	NA
**Corticoid use, n**					
No	25	56%	20	44%	78
Yes	14	44%	18	56%	10
**Alcohol consumption, n**					
No consumption	14	54%	12	46%	30
1–2 units daily	15	45%	18	55%	38
> 2 units daily	0	0%	1	100%	6
Unknown	10	59%	7	41%	14
**Tobacco use, n**					
Previous user	13	68%	6	32%	28
Active user	6	67%	3	33%	5
Non-user	16	41%	23	59%	52
Unknown	4	40%	6	60%	3

NA, not applicable.

**Table 2:** Descriptive data of the extracted teeth in the study and control group.

Characteristics	Oncologic group				Control
Number of extracted teeth, n	218				238
Development of osteonecrosis, n (%)	MRONJ+	%	MRONJ−	%	NA
	63	29%	155	71%	
**Type of teeth, n (%)**					
Incisors and canines	18	30%	43	70%	75
Premolars	14	24%	45	76%	59
Molars	31	32%	67	68%	104
**Region, n (%)**					
Anterior maxilla	8	27%	22	73%	34
Posterior maxilla	22	26%	63	74%	87
Anterior mandible	10	32%	21	68%	41
Posterior mandible	23	32%	49	68%	76
Underlying dental disease, n (%)*					
Nonapparent	3	12%	21	88%	23
Periodontal disease	3	20%	12	80%	31
Endodontic pathology	17	31%	38	69%	62
Combined lesion	40	32%	84	68%	122
**Drug holiday > 60 days, n (%)**					
No	20	36%	35	64%	NA
Yes	43	26%	120	74%	
**Antibiotic prophylaxis, n (%)**					
No	4	50%	4	50%	225
Yes	59	29%	146	71%	13
**Antiseptic mouthwash, n (%)**					
No	2	40%	3	60%	9
Yes	61	29%	147	71%	229
**Use of L-PRF, n (%)**					
No	18	38%	29	62%	228
Yes	45	27%	122	73%	10
**MRONJ worse stage, n (%)**					
Stage 1	32	51%	NA		NA
Stage 2	28	44%	NA		NA
Stage 3	3	5%	NA		NA

Further description of the teeth from the study group that did (MRONJ+) and did not (MRONJ−) develop osteonecrosis is given. NA, not applicable. *Based on the radiographic characteristics, teeth were classified into: periodontally diseased, which had horizontal bone loss, an angular bone defect, or furcation involvement; with endodontic pathology, which presented pulpal caries, widened periodontal ligament space, prosthodontic treatment and concomitant caries, or periapical lesion; and with endodontic-periodontal combined lesions, when presenting characteristics from both groups.

All included patients had at least one administration of zoledronic acid 4 mg or denosumab 120 mg. Eleven patients had treatment with a combination of a bisphosphonate and denosumab. The mean duration of the ARD treatment was 20.8 months (range 1–83). The mean time between pre-operative panoramic radiograph and tooth extraction was of 1.6 months (range 0–12) for the oncologic group and 1.1 months (range 0–9.6) for the control group. While the mean time interval between tooth extraction and post-operative panoramic was 9.6 months (range 0–61.2) for the study and 7.3 months (range 0–70.8) for the control group.

From the 77 oncologic patients, 39 developed MRONJ in 63 tooth extraction sites (50.6% of the study group, 95% CI 0.47–0.70; 28.9% of the extracted teeth, 95% CI 0.23–0.35). From the affected patients, 21 had from one to three tooth extractions and developed MRONJ in all sites, while 18 patients had in average 5.6 tooth extractions (range 2–24) and presented both MRONJ+ and MRONJ− sites. This last group had in average two MRONJ+ sites (range 1–8).

Among the antiresorptive-treated patients, MRONJ+ patients had a longer treatment duration with a mean of 24.9 months, than MRONJ− patients, who had a mean of 16.7 months. Despite this difference, the test result was borderline significant (W = 555, *p* = 0.057). Furthermore, no significant differences were found in the distribution of age (W = 785, *p* = 0.659), gender (X^2^ = 0.659, *p* = 0.417), history of chemotherapy and/or radiotherapy (*p* = 0.679), type and number of ARDs (X^2^ = 3.149, *p* = 0.207; W = 684, *p* = 0.376), duration of corticosteroid use (X^2^ = 0.624, *p* = 0.429), alcohol consumption (W = 500, *p* = 0.394), and tobacco abuse (*p* = 0.115), between those who did and did not develop the pathology.

Regarding the extracted teeth, no significant differences were observed in the type of extracted tooth (W = 4695, *p* = 0.633), region (W = 4866, *p* = 0.969), presence of drug holiday (X^2^ = 1.538, *p* = 0.215), use of L-PRF (X^2^ = 1.762, *p* = 0.184), antiseptic mouthwash (*p* = 0.634), nor prophylactic antibiotics (*p* = 0.239), between the MRONJ+ and MRONJ− sites.

The overall Kappa for interobserver agreement was moderate (0.66), ranging between a fair (0.34) and an almost perfect agreement (0.96) in the periodontal ligament assessment and the presence of endodontic treatment, respectively. The mean Kappa value for the intraobserver agreement was substantial (0.79) for the overall assessment, ranging between a moderate agreement (0.45) in the assessment of lamina dura, and an almost perfect agreement (0.98) in the presence of endodontic treatment.

### Pre- and post-operative parameters: control versus study group

Thickening of the lamina dura was significantly more present in the antiresorptive-treated patients (10%) than in the control group (3%, *p* = 0.003). Significant lower caries and specifically pulpal caries prevalence was also seen in this group (34%) in comparison to the control group (45%, *p* = 0.006).

When looking at the bone pattern surrounding the extraction site postoperatively, the oncologic group had a higher prevalence of a sclerotic (33%) and a heterogeneous pattern (6%, *p* < 0.001), than the control group (20% and 0%, respectively). The visibility of the alveolar socket after the extraction (*p* < 0.001), and when looking at those sites with images taken at least one year postoperatively (*p* < 0.001), were also significantly more prevalent among the patients under ARD. The same was observed for the persistence of the lamina dura (*p* < 0.001). Finally, the visibility of sequester formation was present only in the ARD group (3%, *p* = 0.012).

### Pre- and post-operative parameters: MRONJ+ versus MRONJ−

**Figure 1:**
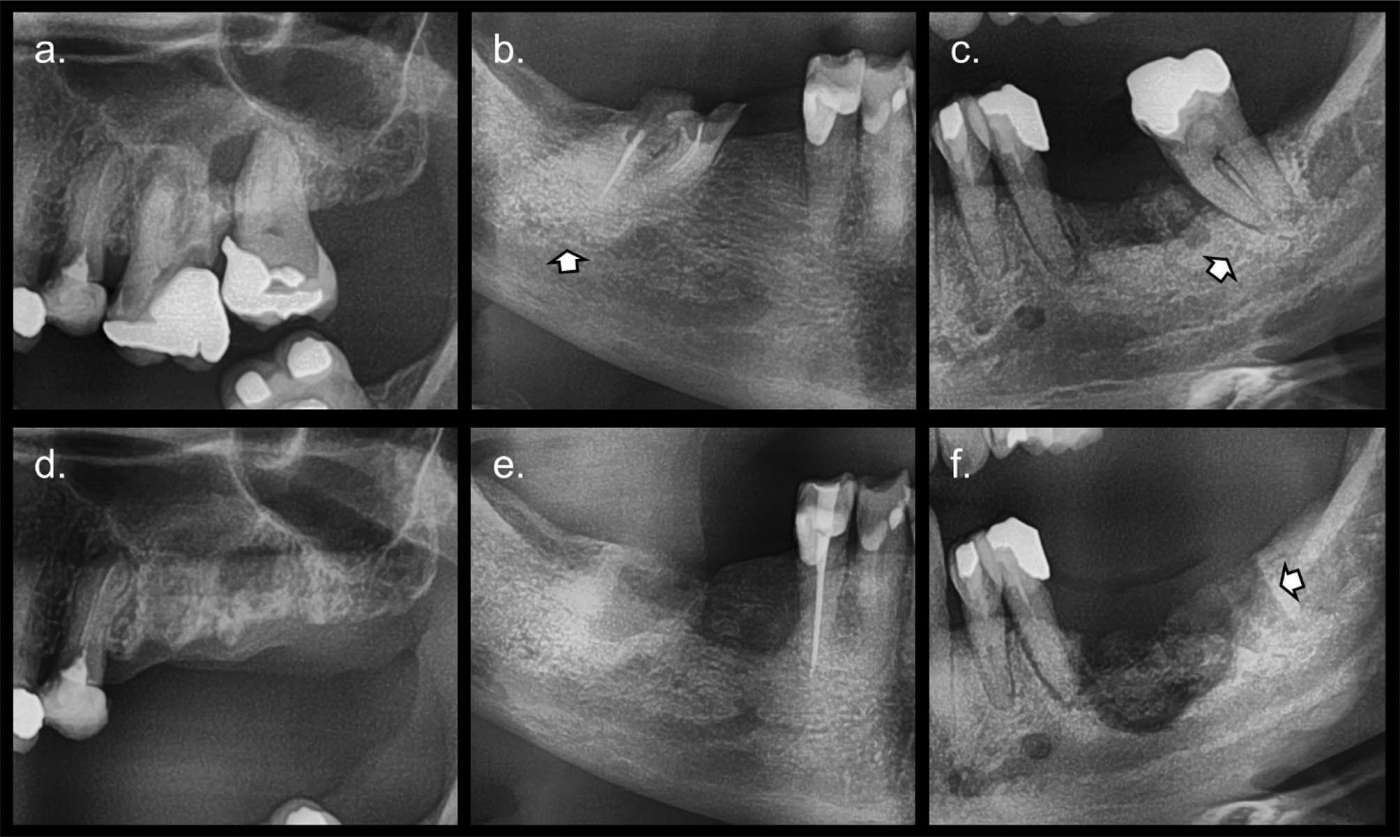
Cropped panoramic images showing pre-operative sites (**a**–**c**) and their respective post-operative evolution (**d**–**f**). Pre-operative images show presence of risk factors, teeth without endodontic treatment and with periapical lesion (**a**, **c**), incomplete endodontic filling in length and presence of caries (**b**), and sclerotic bone pattern (**b**, **c**; white arrow). All extraction sites developed osteonecrosis, showing a heterogenous bone pattern (**d**, **f**), a sclerotic bone pattern (**e**), visible extraction socket (**e**, **f**), persistence of the lamina dura (**f**; white arrow), and sequester formation (**f**).

Teeth that were not treated endodontically developed MRONJ more frequently (35%), than teeth with endodontic treatments (17%). However, when present, an endodontic filling material insufficient in length and/or width increased the chance of the onset of MRONJ (*p* = 0.005). Additionally, 82% of the extracted teeth had either caries, widened periodontal ligament space and/or periapical lesions. From these decayed teeth, 37% without endodontic treatment, 13% with adequate fillings, and 31% with inadequate fillings, developed MRONJ. There was no development of MRONJ in teeth without signs of endodontic infection and presence of endodontic treatment, whether adequate or inadequate. Lastly, a pre-operative bone pattern different than normal increased the likelihood of MRONJ (*p* = 0.005), as 46% of the sclerotic and 67% of the heterogeneous sites developed the pathology. Detailed results are displayed in Table 3 and an illustrative example in Fig. 1.

**Table 3:** Shows the distribution of extraction sites according to pre- and post-operative radiographic characteristics observed in the MRONJ+ and MRONJ− subgroups and in the control group.

Observed parameters		Oncologic group			Control group	
		MRONJ+	MRONJ−	*p*-value	n	*p*-value
**Pre-operative assessment**						
Horizontal bone loss	Absent/initial	23 (26%)	65 (74%)	0.557	99	0.864
	Moderate/severe	40 (31%)	90 (69%)		139	
Angular bone loss	Absent	58 (30%)	136 (70%)	0.493	224	0.070
	Present	5 (21%)	19 (79%)		14	
Furcation involvement	Absent	46 (28%)	117 (72%)	0.835	179	1.000
	Present	17 (31%)	38 (69%)		59	
Periodontal ligament space	Normal	19 (21%)	70 (79%)	0.059	109	0.329
	Widened	44 (34%)	85 (66%)		129	
Lamina dura	Normal	56 (29%)	140 (71%)	0.944	231	*0.003*
	Thickened	7 (32%)	15 (68%)		7	
Root remnant	No	50 (29%)	123 (71%)	1.000	198	0.352
	Yes	13 (29%)	32 (71%)		40	
Periapical lesion	Absent	41 (29%)	102 (71%)	1.000	153	0.846
	Present	22 (29%)	53 (71%)		85	
Endodontic treatment	Absent	51 (35%)	95 (65%)	*0.005*	145	0.539
	Adequate filling	4 (9%)	39 (91%)		54	
	Inadequate filling	8 (28%)	21 (72%)		39	
Prosthodontic treatment	Absent	30 (35%)	56 (65%)	0.155	107	0.274
	Present	33 (25%)	99 (75%)		131	
Caries depth	Absent	32 (26%)	91 (74%)	0.253*	104	0.006*
	Reaches dentine	6 (29%)	15 (71%)		26	
	Reaches pulp	25 (34%)	49 (66%)		108	
Bone pattern preoperative	Normal	40 (24%)	128 (76%)	*0.005*	193	0.273
	Sclerotic	21 (46%)	25 (54%)		44	
	Radiolucent	0 (0%)	1 (100%)		1	
	Heterogenous	2 (67%)	1 (33%)		0	
**Post-operative assessment**						
Bone pattern postoperative	Normal	27 (21%)	104 (79%)	< *0.001*	187	< *0.001*
	Sclerotic	24 (33%)	48 (67%)		47	
	Radiolucent	2 (67%)	1 (33%)		3	
	Heterogenous	10 (83%)	2 (17%)		1	
Alveolar socket	Absent	24 (29%)	60 (71%)	1.000	168	< *0.001*
	Visible	39 (29%)	95 (71%)		70	
Lamina dura	Absent	31 (29%)	76 (71%)	1.000	183	< *0.001*
	Visible	32 (29%)	79 (71%)		55	
Sequestrum formation	Absent	57 (27%)	155 (73%)	< *0.001*	238	*0.012*
	Visible	6 (100%)	0 (0%)		0	
Crater-like defect	Absent	50 (25%)	147 (75%)	*0.001*	223	0.253
	Visible	13 (62%)	8 (38%)		15	

The *p*-value under “oncologic group” describe the results obtained from the comparison of MRONJ+ and MRONJ− sites, while the *p*-value under “control group” describe the results from the comparison of study and control sites. Italic text is marked in those assessments where differences are statistically significant. Results obtained using the exact chi-square/Fisher’s exact test, except for (*), which used the Wilcoxon rank sum test.

A post-operative heterogeneous (83%) and radiolucent bone pattern (67%) was witnessed significantly more at MRONJ+ than MRONJ− sites (*p* < 0.001), while 67% of the sclerotic and 79% of the normal bone patterns were seen in MRONJ− sites. Furthermore, sequester formation was exclusively seen in locations that developed MRONJ. These differences were thus significant (*p* < 0.001). Moreover, crater-like defects were significantly more detected, and when present more extensive, in MRONJ+ sites (62%) in comparison to MRONJ− (< 0.001).

Results of the paired test regarding the bone pattern before and after tooth extraction show a significant difference in the appearance among the oncologic patients (X^2^ = 36.77, df = 6, *p* < 0.001) but not in the control group (X^2^ = 3.27, df = 6, *p* = 0.773). In the study group, it was seen that 38 sites that had initially a normal trabecular pattern showed a sclerotic one after surgery, and four a heterogenous pattern. Moreover, eight sites with a pre-operative sclerotic pattern showed after tooth extraction a heterogeneous one.

Lastly, teeth were classified based on their radiographic characteristics into, periodontally diseased, with endodontic pathology, with endodontic-periodontal combined lesions, or no apparent disease, as described in Table 2. However, no significant differences were found between the control and study group (*p* = 0.161) nor the subgroups MRONJ+ and MRONJ− (*p* = 0.219).

### Within patient analysis: MRONJ+ and MRONJ−

When looking at ARD-patients that had multiple extractions and sites that did and did not develop MRONJ, the assessment of endodontic treatment status (*p* = 0.033) and pre-operative surrounding bone pattern (*p* < 0.001) showed significant results. More specifically, and as mentioned before, a sclerotic bone pattern and absent and inadequate endodontic treatments showed a higher predisposition for the pathology.

### Multivariate analysis of risk factors

**Figure 2:**
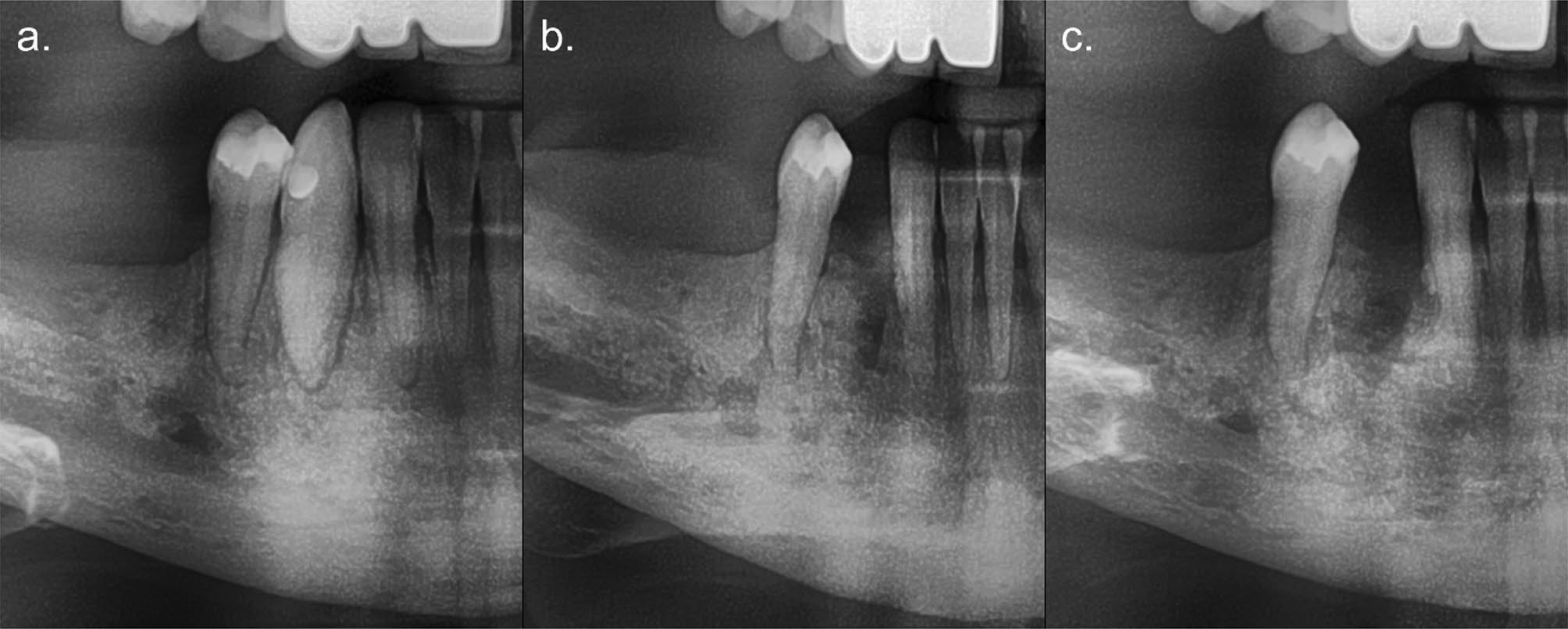
Cropped panoramic radiographs of a 67-year-old female in treatment with denosumab, showing tooth 43 two months before extraction (**a**), and ten (**b**) and eighteen months after (**c**). This site had absence of periapical lesion and angular bone defect, but presence of sclerosis and widening of the periodontal ligament space. Osteonecrosis was diagnosed eight weeks after surgery, as the site did not heal. Persistence of the alveolar socket and sclerotic pattern can be seen postoperatively.

Additionally, the logistic regression model showed significant results in the pre-operative assessment for the variables, endodontic treatment (*p* = 0.019), periapical lesion (*p* = 0.002), surrounding bone pattern (*p* = 0.013), and angular bone defect (*p* = 0.048). However, when performing pairwise comparisons and correcting for multiple testing, only the absence of periapical lesion (OR = 1.78, 95% CI 1.459–2.175, *p* = 0.002) and angular bone defect (OR = 1.72, 95% CI 1.278–2.306, *p* = 0.048), and the presence of a sclerotic bone pattern (OR = 3.45, 95% CI 1.094–10.309, *p* = 0.027), showed significant results (Fig. 2).

## Discussion

Radiographic bone changes in the maxilla and mandible, after the intake of antiresorptive drugs, have been widely reported in the literature even before clinical exposure of bone in the oral cavity. Although, it is unclear whether most of these findings are solely related to the antiresorptive therapy, indicate a potential progress to osteonecrosis, or represent different stages of the necrotic process^15^. In the present study, we aimed to confirm the associations we previously identified in a smaller and different patient cohort, and validate those pre-operative radiologic local risk factors to the development of MRONJ in patients treated with oncologic-related doses undergoing tooth extraction^11^. To our knowledge, this is the first large series to include a pre- and post-operative radiologic assessment of the extraction site.

Prevention in patients who belong to the category “at risk” according the AAOMS^6^ is key to reduce the incidence of this pathology^16–18^. Yet, these strategies are not always timely and dental extractions may become inevitable. Given the need for extractions, it is worth questioning how many of these teeth with pain, associated radiographic changes and absence of bone exposure are classified as "at risk", when in fact they belong to "stage 0". This refers above all to sites that showed clinical post-operative bone exposure and suggests that the pain, initially considered of dental origin, might be due to an undiagnosed osteonecrosis. Validating this point, Nicolatou-Galitis et al. found in such patients the presence of necrotic bone in biopsies obtained at tooth extraction^10^.

In this light, the use of radiographs for diagnostics, treatment, and the identification of risk factors at the extraction site is of utmost importance to plan and understand possible complications. In that sense, panoramic radiographs are widely available in the dental practice environment^19,20^, thus a good starting point for diagnosis and risk assessment. However, for a thorough diagnosis, a three-dimensional method such as computed tomography (CT) and Cone Beam CT is recommended^21^. These tools allow the assessment of the extension of the pathology^22,23^ and to identify incipient lesions like those seen in patients “at risk” or in “stage 0”^15,21,23^. Consequently, new diagnostic staging systems have been proposed using a complement of clinical and radiographic signs^24,25^.

Comparable to what has been described in other studies, patients under ARD, in contrast to the control group, showed significantly more thickening of the lamina dura^11,15,19^. Thickening of the lamina dura and the mandibular cortex, enhancement of the mandibular canal, and trabecular sclerosis are all examples of the sclerosing process that the bone undergoes secondary to antiresorptive treatment. These sclerotic forms have been reported by numerous authors^19,20,26–29^. In fact, Gaêta-Araujo et al. argued that such sclerotic changes in bone might have a better relationship with the antiresorptive therapy per se than a predisposing factor to MRONJ^11^.

In the present sample, sites which initially showed sclerosis seem to have a larger chance of developing osteonecrosis than those with a normal pattern. Besides, bone remodeling led in eight initially sclerotic sites to a postoperative heterogeneous pattern, which in most cases (7/8) were associated to MRONJ, while bone remodeling from a normal to a heterogeneous pattern was less common. Albeit every extracted tooth in sclerotic sites had either periodontal, endodontic, or endodontic-periodontal disease. As discussed by other authors, these sclerotic changes can also be a bony response to local dental infection^11,30,31^. Thus, it is conceivable that sites with sclerotic changes represent a risk factor for MRONJ^32^, but this higher risk could be also due to underlying dental disease, or a combination of both.

Although the results border on significance, oncologic patients with longer ARD treatments and undergoing tooth extractions have a higher chance of developing osteonecrosis, since MRONJ+ patients had a mean treatment of 24 months and MRONJ− of 16 months. These findings align to what is reported in the literature^7–9^. Some authors debate that besides the length of the treatment, the type of drug, whether bisphosphonates or denosumab, differed in their radiographic appearance^22^. However, these findings are described in exposed variants of MRONJ and these differences were not studied in this cohort due to the restricted sample size.

Perhaps the most important findings of the present study are those related to the MRONJ+ group, being that the pathology presented itself significantly more in teeth with absent and incomplete endodontic fillings, and a sclerotic and heterogenous alveolar bone pattern. Furthermore, the most likely combination of radiological characteristics leading to MRONJ was a preoperative sclerotic bone pattern in absence of periapical lesion and angular bone defect. Nevertheless, teeth with these features were not exempt of dental disease, as they had periodontal ligament space widening (79%), radiographic signs of periodontitis (62%), caries (50%), or were root remnants (29%). Therefore, the results of the multivariate analysis may find an explanation in the sample distribution, rather than in the absence of chronic infection and its relationship with MRONJ.

Endodontic treatments have been strongly advocated in patients receiving ARD since the first appearances of the pathology, in order to promote conservative treatments and thus avoid bone trauma^33^. Yet, the present results reveal that the quality of endodontic treatment is an equally important aspect to consider, especially in teeth presenting with signs of infection such as caries, periapical lesions and widening of the periodontal ligament. We saw that decayed teeth with inadequate endodontic treatments exhibited more osteonecrosis than those in the same condition with adequate fillings. Supporting this finding, it has been described that the root-filling technique influences the success of the endodontic treatment^34^. Therefore, it seems a plausible explanation that infected teeth with inadequate endodontic fillings are a greater reservoir of chronic infections than its counterpart.

In terms of post-operative findings, persistence of the alveolar socket is associated with the development of osteonecrosis^19,35^, which in turn is associated with previous bone sclerosis at the site^35^. In addition, osteolytic changes are associated with progression to exposed osteonecrosis^36^ and sequestrum formation^35^. In our sample, a heterogenous bone pattern, sequestrum formation, persistence of the alveolar socket, and crater-like defect were seen significantly more in the MRONJ+ group. Still, sequestrum appears to be a pathognomonic feature of MRONJ, as it appeared only in MRONJ+ sites. Though, knowing that this pathology manifests itself early with different bone patterns and absence of clinically exposed bone^21^, it may be that some of these findings are a subclinical form of osteonecrosis^36^. This observation stresses the value of diagnostic imaging and a closer follow-up.

Among the limitations of this study, we found those inherent to its retrospective nature, namely, lack of data recording in the patient file, surgical variability, different ARD treatment and drug holiday duration, polypharmacy and comorbidity factors, midst other variables that could not be fully controlled. In addition, the prevalence of MRONJ in this sample, 51% of the patients and 29% of the extracted teeth, seems higher than what is reported in the literature (0.5–4.6% of the patients^37^), but comparable to a prior Belgian study^38^, where the risk of MRONJ increased to 20% after tooth extractions. Lastly, the timing of the exposure to the radiograph before and after the extraction was not homogeneous either, giving room for changes in the appearance of the assessment perceptible to the observer.

The clinical relevance of this study relies on the need for diagnostic imaging prior to tooth extraction in oncologic patients under ARD. Specially to identify a sclerotic and heterogeneous preoperative bone pattern, which in this study we consider as high risk for MRONJ, but perhaps they are already an early stage of osteonecrosis without bone exposure. Likewise, follow-up images can indicate the onset of MRONJ with the presence of a heterogeneous bone pattern, persistence of the alveolar socket, a crater-like defect, and bone sequester. Moreover, when the high-risk local factors are recognized, a treatment plan that involves a closer follow-up after tooth extraction(s), the use of prophylactic antibiotics^39,40^, antiseptic mouthwash^41^, and L-PRF for MRONJ prevention^39,42,43^, are recommended. As a word of caution, we suggest careful interpretation of our results, as the purpose of the univariate and multifactorial statistical analysis was to identify variables (or combinations of) that have a relationship with the development of osteonecrosis, rather than to establish causality. Further prospective studies are necessary to confirm the present findings.

## Conclusion

Osteonecrosis may be anticipated upon recognition of the following associations described in the present study: teeth with absent and incomplete endodontic fillings with caries, widened periodontal ligament space and/or periapical lesions, and a sclerotic and heterogeneous preoperative alveolar bone pattern, in patients with longer ARD treatments. Most findings highlight the relevance of local infectious factors in the development of this pathology. Early identification of these features encourages a patient-specific decision making to take preventive measures during the treatment, giving importance to the quality of the conservative treatment and chronic dental infections in sites needing extraction in oncologic patients under ARD treatment.
